# Short-Term Outcomes of Dual Versus Single Antiplatelet Therapy Following Popliteal and Infrapopliteal Endovascular Therapy: Data From Dutch Chronic Lower Limb-Threatening Ischemia Registry (THRILLER)

**DOI:** 10.1177/15266028241312356

**Published:** 2025-01-22

**Authors:** Emilien C. J. Wegerif, Michael J. Nugteren, Isa F. van Galen, Constantijn E. V. B. Hazenberg, Michiel A. Schreve, George P. Akkersdijk, Bram Fioole, Maurice Pierie, Olaf Schouten, Daniel A. F. van den Heuvel, Olaf J. Bakker, Jan-Willem Hinnen, Bart A. N. Verhoeven, Jan M. M. Heyligers, Maarten K. Dinkelman, Gert J. de Borst, Çağdaş Ünlü

**Affiliations:** 1 Department of Vascular Surgery, University Medical Center Utrecht, Utrecht, The Netherlands; 2 Department of Vascular Surgery, Northwest Hospital Group, Alkmaar, The Netherlands; 3 Department of Vascular Surgery, Maasstad Hospital, Rotterdam, The Netherlands; 4 Department of Vascular Surgery, Isala Hospital, Zwolle, The Netherlands; 5 Department of Radiology, St. Antonius Hospital, Nieuwegein, The Netherlands; 6 Department of Vascular Surgery, St. Antonius Hospital, Nieuwegein, The Netherlands; 7 Department of Vascular Surgery, Jeroen Bosch Hospital, ‘s-Hertogenbosch, The Netherlands; 8 Department of Vascular Surgery, Elisabeth-TweeSteden Hospital, Tilburg, The Netherlands

**Keywords:** chronic limb-threatening ischemia, antiplatelet therapy, antithrombotic therapy, infrapopliteal occlusive disease, peripheral artery disease, endovascular therapy

## Abstract

**Objective:**

There is a lack of consensus regarding the optimal antithrombotic therapy (ATT) after popliteal and infrapopliteal (PIP) endovascular therapy (EVT). Currently, dual antiplatelet therapy (DAPT) for 3 months and single antiplatelet therapy (SAPT) are the most prescribed regimens in the Netherlands. Thus far, no randomized comparison has been performed on the optimal ATT approach. Therefore, this study compared the efficacy and safety of 3-month DAPT with SAPT following PIP EVT.

**Design:**

Retrospective analysis of prospectively collected data from a multicenter registry.

**Methods:**

The Dutch chronic lower limb-threatening ischemia registry (THRILLER) collected prospective data on patients enrolled between January 2021 and October 2023. As for ATT, only patients prescribed antiplatelet therapy (APT), were included in this analysis. The primary efficacy outcome was a composite of 3-month major adverse cardiovascular events (MACEs, ie, myocardial infarction, cerebrovascular event, cardiovascular death), major adverse limb events (MALEs, ie, major amputation, reintervention), and non-cardiovascular death. Secondary efficacy outcomes were 3-month MACE, MALE, and all-cause mortality. The primary safety outcome was major bleeding according to the ‘Thrombolysis In Myocardial Infarction’ (TIMI) classification. Descriptive statistics and Cox proportional hazard models were applied.

**Results:**

In total, 460 of 840 THRILLER patients used DAPT or SAPT as ATT and were therefore included in the analysis. Of these, 322 (70%) received DAPT and 138 (30%) received SAPT. In total, 73 (15.9%) primary efficacy outcomes were observed of which 21 (15.2%) events in the SAPT group and 52 (16.1%) events in the DAPT group. No significant differences were observed between SAPT and DAPT for the primary efficacy outcomes or any of the secondary efficacy outcomes. In both groups, one case of major bleeding was observed.

**Conclusion:**

The findings suggest that 3 months of DAPT is not superior to SAPT. A well-powered randomized trial is warranted to assess the efficacy and safety of post-procedural DAPT in chronic limb-threatening ischemia (CLTI) patients undergoing PIP EVT.

**Clinical Impact:**

This manuscript reports on the efficacy and safety outcomes of 3 months of DAPT versus SAPT, which are commonly chosen therapies following popliteal and infrapopliteal endovascular therapy. No significant difference was found between the two groups regarding major adverse cardiovascular events, all-cause death, major amputation, or major bleeding. Therefore, 3 months of DAPT does not seem superior to SAPT. These results suggest that SAPT appears to be a sufficient alternative when considering 3 months of DAPT. Further research should verify these outcomes and focus on the efficacy and safety of prolonged DAPT suppletion after endovascular therapy.

## Introduction

The treatment of popliteal and infrapopliteal (PIP) atherosclerotic lesions in patients with chronic limb-threatening ischemia (CLTI) is challenging since these lesions are associated with severe calcification, small vessel diameter, longer lesion length, and multilevel atherosclerosis.^1^ Subsequently, these patients have twice the risk of major adverse cardiovascular events (MACEs) and restenosis compared with patients treated above the knee.^2^ Therefore, after PIP endovascular therapy (EVT), secondary antithrombotic therapy (ATT) is crucial to reduce the increased risk for major adverse limb events (MALEs) and MACE.^3,4^ Defining the optimal ATT strategy after this treatment remains challenging due to lacking evidence. Despite evidence for continuing best medical treatment after EVT, which includes single antiplatelet therapy (SAPT), there is a trend to intensify the ATT for short term after EVT.^5–8^

Within the field of interventional cardiology, this trend has already been proven and, therefore, after percutaneous coronary interventions (PCIs) with drug-eluting technology, dual antiplatelet therapy (DAPT) for 1 to 12 months (depending on the manifestation of coronary artery disease [CAD]) is the golden standard.^9,10^ However, PCI and EVT differ regarding the location, vessel length, and device technologies, with PCI being characterized by the predominance of drug-eluting devices and endovascular revascularization (EVR) by a wider range of devices. Therefore, the optimal ATT after PCI cannot simply be adapted to EVT without confirmatory evidence. The lack of evidence for the optimal ATT strategy leads to different recommendations per guideline. The Dutch guideline suggests clopidogrel as SAPT, the Society for Vascular Surgery (SVS) recommends short-term DAPT for 1 to 6 months and the European Society of Vascular Surgery (ESVS) advises DAPT for 6 to 12 months.^5,6^ Surveys among vascular surgeons and interventional radiologists show that after below-the-knee EVTs 55% to 73% of the patients were prescribed DAPT and in 25% to 43% SAPT following below-the-knee EVT.^7,11^ If DAPT was prescribed, the most chosen duration was 3 months since previous studies have shown that most adverse events occur in these months after EVT in CLTI patients.^2,7,12–14^ Dual antiplatelet therapy may be associated with an increased risk of major bleeding and, therefore, the ESVS guideline included a separate recommendation to take the bleeding risk into account.^8^ However, patients undergoing PIP EVT frequently have multiple comorbidities, which together easily result in a high-risk class for major bleeding.^15^ Considering the challenging balance between the high risk of MACE and MALE following PIP EVT, the high risk of major bleeding, and the preference for 3 months of DAPT among surgeons, additional evidence on the efficacy and safety of short-term DAPT is warranted. This study aimed to examine whether DAPT compared with SAPT, reduces the risk of short-term (ie, 3 months) MACE, MALE, and non-cardiovascular mortality in patients undergoing PIP EVT, based on the data of the prospective registry.^16^

## Materials and Methods

### Data Source

The Dutch chronic lower limb-threatening ischemia registry, THRILLER, is an ongoing, Dutch, multicenter, observational registry, enrolling CLTI patients who underwent popliteal or infrapopliteal EVT across 7 high-volume vascular centers (NTR ID: NL 9192; URL: https://trialregister.nl/trial/9192).^16^ Written informed consent was obtained from all patients prior to EVT to retrieve data from patient records. This article contains a retrospective analysis of prospectively collected data from a prospective multicenter registry. Data are collected every 4 months from electronic patient records using the web-based reporting system Castor.^16^ Follow-up was mandatory at 6 to 8 weeks and 12 months following EVT. Additional outpatient visits were optional. Due to this design and broad inclusion criteria, it is a large well-phenotyped data set with <1% missing data.

### Study Population

All patients in THRILLER were aged ≥18, diagnosed with Rutherford classes 4 to 6 and underwent (at least) popliteal and/or infrapopliteal EVT.^16^ Patients were excluded if they would undergo EVT for acute limb ischemia (ALI), distal embolization, or nonatherosclerotic arterial disease or aneurysmal disease. A total of 840 patients were included between January 2021 and October 2023.

For this subgroup analysis, all patients were considered eligible for inclusion unless they were using ATT other than a P2Y12 inhibitor (ie, clopidogrel, ticagrelor, prasugrel) and/or acetylsalicylic acid (ASA) at discharge, had no recorded ATT information, or had a history of coagulopathy.

### Efficacy and Safety Objectives

The primary efficacy objective was whether and to what extent 3-month DAPT is superior to SAPT regarding the composite of 3-month MACE (ie, myocardial infarction, cerebrovascular event, and cardiovascular death), MALE (ie, major amputation, reinterventions, such as EVT, surgical bypass, and thrombolysis), and non-cardiovascular death. Secondary efficacy objectives were whether and to what extent 3-month DAPT was superior to SAPT regarding MACE, MALE, and all-cause mortality. Moreover, differences between the primary efficacy and MALE outcomes between DAPT and SAPT groups within the infra-inguinal stented and infra-inguinal non-stented populations were assessed. The primary safety outcome was major bleeding following the ‘Thrombolysis In Myocardial Infarction’ (TIMI) classification.^17^

### Data Collection

Patient characteristics, including sex, age, comorbidities, antiplatelet therapy (APT) prescription, and follow-up data, including efficacy and safety outcomes, were obtained. Outcomes were assessed up to 3 months following EVT. Subsequently, patients were categorized into 2 groups based on their discharge APT regimens: (1) individuals who received SAPT with either ASA or P2Y12 inhibitors and (2) DAPT users with P2Y12 inhibitors and ASA.

### Statistical Analysis

Continuous variables were presented as mean with standard deviation (SD) for normally distributed data and as median with interquartile range (IQR) for non-normally distributed data. Categorical variables were shown as counts and percentages. The baseline characteristics between the DAPT and SAPT groups were compared using the independent samples *t* test for normally distributed data and the Mann-Whitney *U*-test for non-normally distributed data. Differences between categorical variables were assessed using the chi-square test or Fisher’s exact test, if appropriate.

Kaplan-Meier curves were used to demonstrate cumulative survival for all outcomes, while log-rank tests were utilized to compare differences in survival probabilities over time between DAPT and SAPT. Cox proportional hazard models were used to estimate the risk of clinical outcomes between the SAPT and DAPT groups, taking into account the reasonable assumption of proportional hazards. In the multivariate Cox regression analysis, risk factors for MACE and MALE, American Society of Anesthesiologists Physical Status Classification, and Rutherford classification were included.^5^ These variables were extracted from similar studies.^7^

Results were presented as a hazard ratio (HR) with a 95% confidence interval (CI), and significance was determined at p values<0.05. IBM SPSS Statistics software (IBM, Armonk, NY, USA) was used for statistical analysis.

## Results

### Study Population

**Figure 1.**
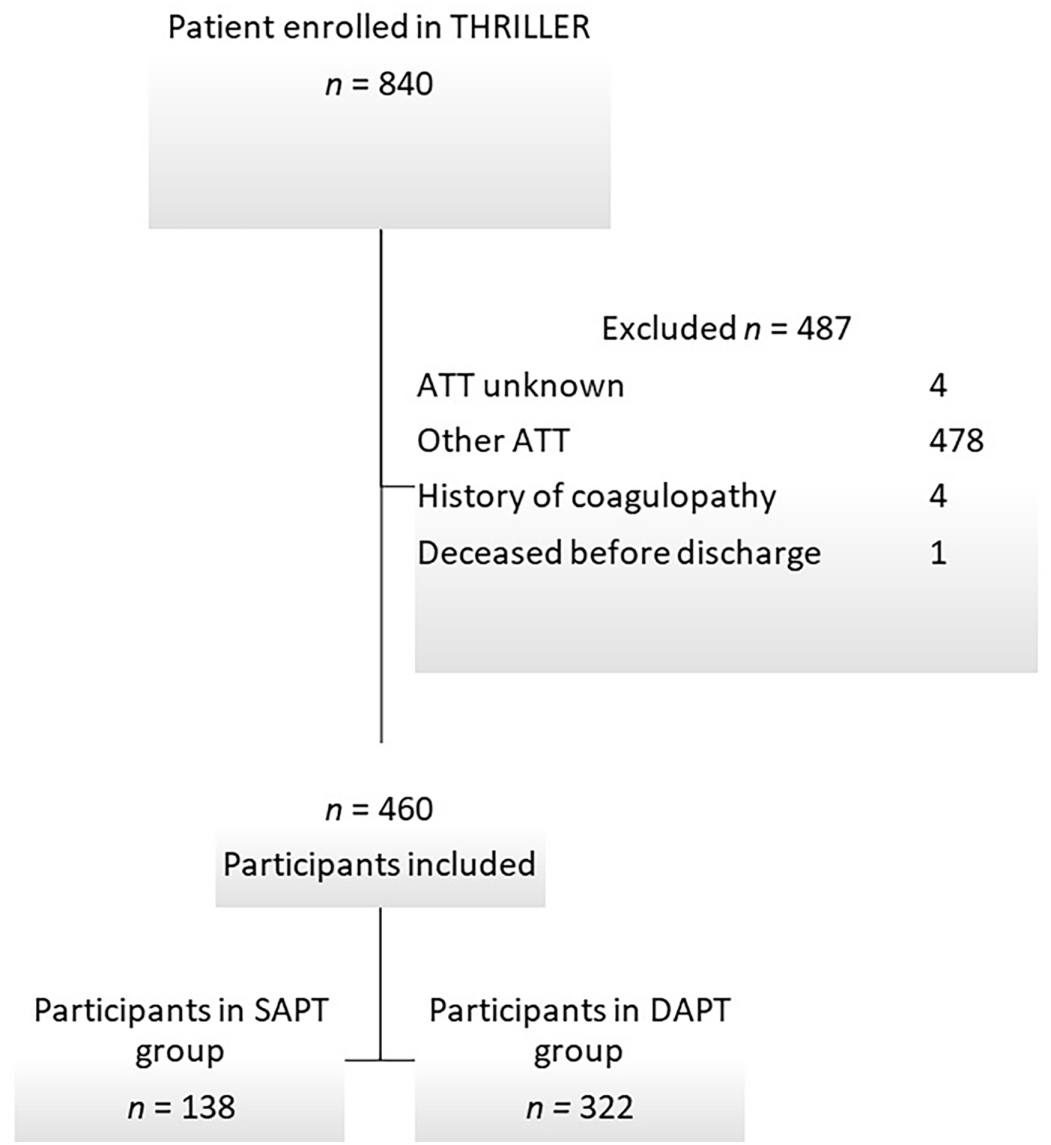
Flowchart of the inclusion process.

In total, 840 patients were included in THRILLER and 487 were excluded from this analysis mainly due to other ATT, including DAPT or SAPT plus anticoagulation (Figure 1). Reasons for other ATT were mainly atrial fibrillation (n=311, 37%), followed by embolism/(floating)thrombus/thrombosis, heart-valve stenosis or replacement, implantable cardioverter defibrillator, history of bypass surgery, and antiphospholipid syndrome. After excluding patients who did not meet the inclusion criteria for this sub-analysis, 460 patients remained (Figure 1). Among them, 322 (70%) participants were prescribed DAPT and 138 (30%) patients received SAPT. The study population had a median age of 74 (IQR=13), was predominately male (71.1%), and contained mostly Rutherford category 5 patients (80.7%) with WIfI class 4 (34%). All baseline characteristics were balanced between the 2 groups except for age and cardiovascular diseases (CVDs), which were both slightly higher in the SAPT group (p=0.038 and p=0.003, respectively; Table 1). In total, 147 patients received an infra-inguinal stent whereafter 18 patients received SAPT and 129 patients DAPT.

**Table 1.** Patient Characteristics at Baseline.

Characteristic	SAPT (n=138)	DAPT (n=322)	p value
Age (IQR)	75 (16)	73 (13)	0.038^a^
Tobacco use	102 (73.9)	251 (78.0)	0.163
Diabetes mellitus	89 (64.5)	213 (66.1)	0.732
Hyperlipidemia	122 (88.4)	283 (87.9)	0.363
Hypertension	120 (87.0)	283 (87.9)	0.781
CAD	65 (47.1)	148 (46.0)	0.822
CeVD^b^	38 (27.5)	78 (24.2)	0.003^a^
Renal failure^c^	9 (6.5)	10 (3.1)	0.092
Dialysis-dependent renal failure	5 (3.6)	12 (3.7)	0.957
*ASA class*			
2	17 (12.3)	37 (11.5)	0.781
3	101 (73.2)	230 (71.4)	
4	20 (14.5)	55 (17.1)	
*Rutherford category*			
4	24 (17.4)	54 (16.8)	
5	112 (81.2)	259 (80.4)	0.756
6	2 (1.4)	9 (2.8)	
WIfI classification			
1	21 (15.2)	47 (14.6)	
2	19 (13.8)	55 (17.1)	
3	33 (23.9)	79 (24.5)	0.916
4	48 (34.8)	110 (34.2)	
Not specified	17 (12.3)	31 (9.6)	

Data are presented as n (%) or median (interquartile range).

Abbreviations: APT, antiplatelet therapy; ASA, American Society of Anesthesiologists; CAD, coronary artery disease; CeVD, cerebrovascular disease; DAPT, dual antiplatelet therapy; eGFR, estimated glomerular filtration rate; IQR, interquartile range; SAPT, single antiplatelet therapy.

a Statistically significant difference.

b Myocardial infarction, angina pectoris, or post-coronary revascularization.

c eGFR<30 mL/min/1.73 m^2^.

If SAPT was prescribed, clopidogrel was prescribed in 66.2% of cases (Table 2). Regarding DAPT, clopidogrel plus ASA was mostly chosen (94.1%). The duration of DAPT varied between 1, 3, and 12 months. The majority of the DAPT duration was unspecified since the follow-up up to 3 months was used, however, 9.9% of the DAPT-prescribed patients received DAPT for 1 month.

**Table 2.** Post-operative Antiplatelet Therapy.

APT	SAPT (n=138)	DAPT (n=322)
Clopidogrel	92 (66.5)	
ASA	41 (29.7)	
Ticagrelor	0 (0.0)	
Prasugrel	0 (0.0)	
Clopidogrel+ASA		304 (94.4)
Ticagrelor+ASA		8 (2.5)
Prasugrel+ASA		1 (0.3)
Unknown	5 (3.6)	9 (2.8)

Data are presented as n (%).

Abbreviations: APT, antiplatelet therapy; ASA, acetylsalicylic acid; DAP, dual antiplatelet therapy; SAP, single antiplatelet therapy.

### Efficacy Outcomes

**Figure 2.**
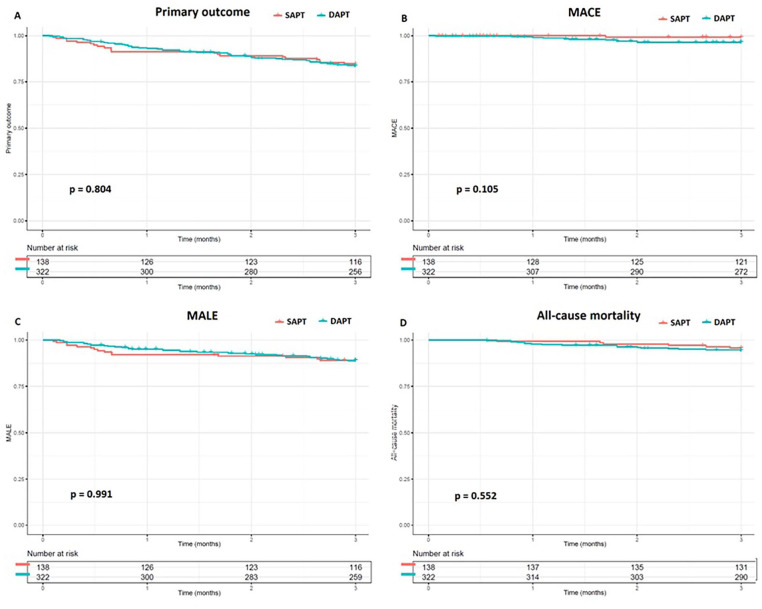
Kaplan-Meier curve up to 3 months following the EVT. (A) primary endpoint, (B) Major Adverse Cardiovascular Events, (C) Major Adverse Limb Events, (D) All-cause mortality.

At 3-month follow-up, 73 (15.9%) events met the criteria of the primary outcome, consisting of MACE, MALE, and all-cause death: 21 (15.2%) events in the SAPT group and 52 (16.1%) events in the DAPT group (Table 3). The Kaplan-Meier curve showed no significant difference at 3-month follow-up between DAPT and SAPT (p=0.804, Figure 2A).

**Table 3.** Incidence and Log-Rank Test of 3-Month Post-Operative Clinical Outcomes.

Outcome	Total (n=460)	SAPT (n=138)	DAPT (n=322)	p value^a^
Primary endpoint (%)	73 (15.9)	21 (15.2)	52 (16.1)	0.804
MACE (%)	12 (2.6)	1 (0.7)	11 (3.4)	0.105
MALE (%)	50 (10.9)	10 (7.2)	19 (5.9)	0.991
All-cause mortality (%)	24 (5.2)	6 (4.3)	18 (5.6)	0.552

Data are presented as n (%).

Abbreviations: MACE, major adverse cardiovascular events, MALE, major adverse limb event, SAPT, single antiplatelet therapy, DAPT, dual antiplatelet therapy.

a The p values from the log-rank test are displayed.

Major adverse cardiovascular event was observed in 12 (2.6%) patients: 1 (0.7%) event in SAPT group and 11 (3.4%) events in DAPT group (Table 3). No significant difference was found between DAPT and SAPT (p=0.105, Figure 2B).

A total of 50 (10.9%) events met the criteria of MALE: 29 events were major amputation and 21 events were reintervention. Of these events, 15 (10.9%) were registered in the SAPT group and 35 (10.9%) in the DAPT group (Table 3). No significant difference was found at 3 months between DAPT and SAPT (p=0.991, Figure 2C).

At 3-month follow-up, 24 (5.2%) patients deceased of which 6 (4.3%) patients received SAPT and 18 (5.6%) DAPT (Table 3). No significant difference was found between DAPT and SAPT (p=0.552, Figure 2D).

**Figure 3.**
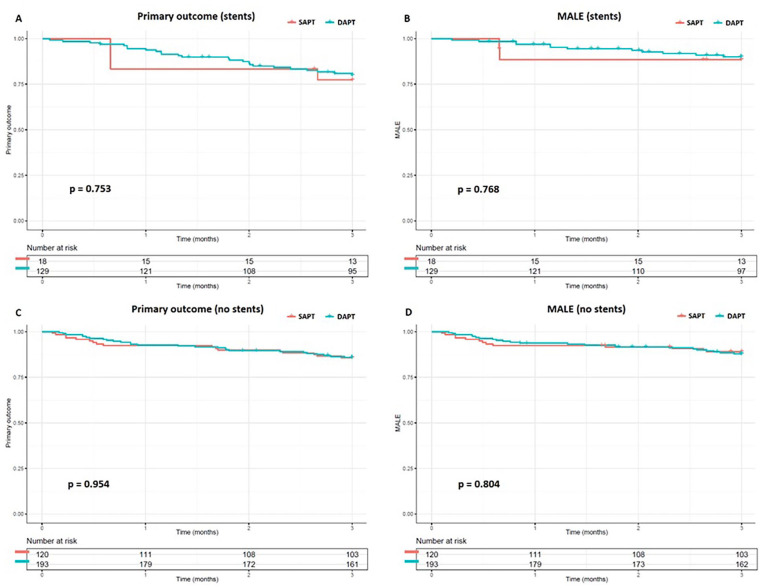
Cumulative Kaplan-Meier estimate of the primary outcome and major adverse limb events (MALE) in the group with infra-inguinal stenting (A, B) and no infra-inguinal stenting (C, D).

For the primary and secondary outcomes, no significant differences were found when adjusting for confounders and covariates at any given time between DAPT and SAPT groups (Table 4). Since clopidogrel as SAPT, and clopidogrel plus ASA as DAPT are most commonly used, the statistics were repeated with only these cases. No significant difference was found. Finally, no difference was found between SAPT and DAPT in the primary efficacy (77.4% vs 80.0%, respectively, p=0.753) and MALE (88.5% vs 90.0%, respectively, p=0.768) outcomes in the infra-inguinal stented population (Figure 3). Nor was any difference found between the primary efficacy (p=0.954) and MALE (p=0.804) outcome in the infra-inguinal non-stented population.

**Table 4.** Cox Regression Analysis of 3-Month Post-Operative Clinical Outcomes.

Outcome	Univariate HR (95% CI)	p value	Multivariate HR (95% CI)	*p* value
Primary endpoint	1.066 (0.642–1.770)	0.805	1.045 (0.806–1.355)	0.739
MACE	4.656 (0.601–36.061)	0.141	N/A^a^	N/A^a^
MALE	0.997 (0.544–1.825)	0.991	1.104 (0.811–1.504)	0.529
All-cause mortality	1.322 (0.525–3.331)	0.554	1.257 (0.499–3.169)	0.627

Abbreviations: CI, confidence interval; HR, hazard ratio; MACE, major adverse cardiovascular event; N/A, not applicable.

a Since the SAPT group has 1 event, a multivariate Cox regression would lead to instability in estimates and is therefore not performed.

### Safety Outcome

At 3-month follow-up, only 2 major bleeding events following the TIMI classification, were collected. One participant (0.7%) suffered from a hemorrhagic stroke under SAPT (clopidogrel) and 1 participant (0.3%) suffered from acute bleeding 16 days after thromboendarterectomy under DAPT (clopidogrel plus ASA) after which he/she deceased.

## Discussion

This study assessed real-world short-term outcomes in CLTI patients who received SAPT or DAPT following PIP EVT, based on a prospective Dutch multicenter registry “THRILLER.” These analyses showed that, up to 3 months after PIP EVT, DAPT was not associated with a significantly reduced risk of MACE, MALE, or non-cardiovascular mortality, compared with SAPT in CLTI patients. Nor did it increase the risk of major bleeding. Noteworthy is two third of this cohort received DAPT and that the primary outcome is mostly driven by the MALEs. Finally, the prescription of ticagrelor in de DAPT group (2.5% of the prescriptions) since the ticagrelor does not add any benefit over clopidogrel.^18^ Three-month follow-up was chosen since previous studies have shown that most adverse events occur in these months after EVT in CLTI patients.^2,7,12–14^

Currently, no golden standard exists regarding the optimal ATT strategy for CLTI patients after PIP EVT due to heterogeneous and limited evidence, which leaves the final choice at the discretion of the treating specialist.^5,6,8^ Currently, VOYAGER is the only large-scale trial for post-intervention ATT in peripheral artery disease (PAD) patients. The researchers concluded that dual pathway inhibition effectively reduces MACE and MALE compared with ASA.^19^ However, sub-analyses show no significant advantage for CLTI patients nor patients undergoing EVT, which does not make it an “end-of-discussion” trial.

The trend toward (3 months) DAPT following (below-the-knee) EVTs has also been observed in our cohort.^2,7,11^ This preference is not fully understood. This might be due to the general high risk of atherothrombotic events in this population since the baseline characteristics were well-balanced between the SAPT and DAPT groups, except for age and CVD. The DAPT group was significantly younger, however, with a median age of 75 versus 73 years, this seems not clinically relevant. Recent research suggests that patients aged 80 and above are associated with an increased risk of bleeding that could play a role.^15^ Concerning CVD, the difference of 27.5% (SAPT) versus 24.2% (DAPT), neither seems clinically relevant. These well-balanced characteristics might result from insufficient evidence for either APT, leaving the rationale behind the choice of ATT mainly unknown.^11^

The preference for DAPT may be extrapolated from evidence in the field of PCI. After extensive research, DAPT remains the cornerstone of secondary prevention of stent thrombosis and atherothrombotic events after PCI.^9,10^ The duration of DAPT differs depending on the manifestation of the disease and guidelines. Hence, multiple studies show that after PCI, 3 months of DAPT compared with 12 months of DAPT is associated with lower major bleedings without increasing the risk of MACE.^20–23^ In addition, since the beneficial effect of DAPT on atherothrombotic events is proven to extend beyond the treated coronary vessel, it may also extend to CLTI patients undergoing PIP EVT.^24,25^ However, research outcomes regarding APT have proven to differ between CAD and PAD patients.^18,26^ Consequently, we cannot be fully confident that DAPT therapy will achieve similar results in CLTI patients. Therefore, cardiology data cannot be extrapolated, hence data extrapolation should be done with care.

In the literature, there is some evidence that DAPT is superior to SAPT in CLTI patients. Three APT registries included, among others, a substantial group of patients with CLTI after EVT (30% up to 100%).^12–14^ These registries showed heterogeneous outcomes on whether and to what extent DAPT is beneficial compared with ASA concerning long-term follow-up (3 up to 5 years): One registry reported on MACE and found a 35% relative risk reduction (RRR) for DAPT.^12^ All 3 registries reported on mortality; the results differed between no statistical difference,^14^ 6% RRR,^13^ and 45% RRR^12^ in the DAPT group. Two registries reported major amputation, 1 registry found no difference^12^ and the other found 74% RRR^14^ for the DAPT group. Major bleeding was mentioned by 1 registry, but no significant difference was found.^14^ However, it is noticeable that concerning the survival figures of all registries, DAPT and SAPT are consistently close to each other at 3 months and, therefore, do not seem to be significantly different, which corresponds with our findings.^12–14^

The rate of major bleeding according to the TIMI classification was only 0.7% and 0.3% in the SAPT and DAPT groups, respectively, which suggests that short-term DAPT does not give an increased risk of short-term major bleeding complications. Therefore, short-term intensified APT, that is, DAPT, does not seem to matter regarding short-term safety. In comparison, the EUCLID (Examining Use of Ticagrelor in Peripheral Artery Disease) trial observed major bleedings in 1.6% in both the clopidogrel and ticagrelor groups at a median follow-up of 30 months.^18^ The CHARISMA (Clopidogrel for High Atherothrombotic Risk and Ischemic Stabilization, Management, and Avoidance) trial observed major bleedings in 1.7% in both the DAPT and ASA groups at a median follow-up of 26 months.^27^

The main limitation of this study is that selection bias may have occurred since only THRILLER patients who were prescribed solely DAPT or SAPT were included, leading to the exclusion of almost half of the registered patients including patients with DAPT or SAPT plus anticoagulation. Moreover, since data are obtained every 4 months However, this study is to our knowledge, the largest (non-randomized) study reporting on short-term DAPT versus SAPT after PIP EVT. In addition, center-specific or physician-specific preferences for DAPT or SAPT may have influenced outcomes. Finally, these analyses did not consider lesion-specific characteristics, such as lesion length and degree of calcification, because during PIP EVT frequently multiple lesions are being treated and no standardized, nor accepted methodology exists to quantify this.

## Conclusion

The results suggest that short-term DAPT does not offer additional risk reduction for short-term adverse ischemic events and all-cause mortality in this registry-based CLTI population. However, it might not lead to a higher risk of major bleeding either. An adequately powered, randomized trial is needed to clarify the efficacy and safety of DAPT in CLTI patients undergoing PIP EVT.
